# Trophic networks improve the performance of microbial anodes treating wastewater

**DOI:** 10.1038/s41522-019-0100-y

**Published:** 2019-09-27

**Authors:** Christin Koch, Katharina J. Huber, Boyke Bunk, Jörg Overmann, Falk Harnisch

**Affiliations:** 1 Helmholtz-Centre for Environmental Research, Department of Environmental Microbiology Permoserstraße 15, 04318 Leipzig, Germany; 2 Leibniz Institute DSMZ—German Collection of Microorganisms and Cell Cultures, Inhoffenstraße 7B, 38124 Braunschweig, Germany; 3 Department of Life Sciences, Braunschweig University of Technology, Braunschweig, Germany; 4 Present Address: Global Innovation Cosmetic Ingredients, Symrise AG, Mühlenfeldstraße 1, 37603 Holzminden, Germany

**Keywords:** Applied microbiology, Environmental microbiology

## Abstract

Microbial anodes represent a distinct ecological niche that is characterized mainly by the terminal electron acceptor, i.e., the anode potential, and the substrate, i.e., the electron source. Here, we determine the performance and the biofilm community of anode microbiomes while using substrates of increasing complexity (organic acids or organic acids and sugar or real domestic wastewater) to mimic different, practically relevant, trophic levels. α-Diversity values increased with substrate complexity. In addition, the higher abundance value of *Deltaproteobacteria* in the biofilms corresponds to higher reactor performance (i.e., COD removal, current density, and Coulombic efficiency). In reactors exploiting real wastewater, the diversity of the planktonic microorganisms was only little affected. Microbiome network analysis revealed two important clusters for reactor performance as well as performance-independent pathogen-containing clusters. Interestingly, *Geobacter* was not found to be integrated in the network underlining its outstanding individual ecological role in line with its importance for the efficiency of the electron harvest for all reactors. The microbiome analysis of different trophic levels and their temporal development from initial colonization to stable treatment demonstrate important principles for the implementation of microbial anodes for wastewater treatment.

## Introduction

Complex microbial communities in technical systems, the so-called reactor microbiomes, form the foundation of environmental biotechnology. Wastewater treatment plants (WWTP) represent the most prominent and best established type of this technology.^1^ Microbiomes in WWTP transform nitrogen, carbon, and phosphorous compounds, and thereby enable sustainable water purification.^2^ Due to their diversity, stability, and flexibility, reactor microbiomes form an unprecedented microbial resource for WW treatment and valorization. Domestic and industrial WW treatment is usually performed in aerobic and anaerobic phases for the oxidation of organic compounds and the denitrification, respectively, of which aeration is the most energy-demanding process. This high-energy demand of aerobic treatment is caused by the high chemical energy content of domestic WW of about 7 kJ L^−1^, and hence the requirement of sufficient oxygen supply for its microbial oxidation.^3^

Microbial electrochemical technologies (MET) offer a sustainable alternative for WW treatment, as they allow combining the oxidation of organic compounds with the generation of electricity,^4^ and thus utilization of the high chemical energy content of domestic WW. In general, MET provide a technology platform that links microbial metabolism, specifically the turnover of metabolic redox carriers like NAD(P)H/H^+^ or quinones, with the flow of electrons to or from an electrode.^5,6^ In recent years, a plethora of MET-based applications ranging from biosensors via water desalination to the synthesis of chemicals has been evolved.^7,8^ The most mature field of application for MET is the treatment and valorization of WW for electric power harvest and hydrogen production; first demonstration units are in the cubic-meter scale.^9,10^

In these MET reactors, anode microbiomes are the key component. Microorganisms oxidize the organic and inorganic WW constituents and transfer the liberated electrons to the anode which serves as a solid terminal electron acceptor (TEA). This extracellular electron transfer (EET) can take place by direct and indirect means^4,11^ and was extensively studied not only in pure cultures of members of the *Geobacteraceae* (e.g., *Geobacter sulfurreducens* PCA^T^ ^12^) and of the *Shewanellaceae* (e.g., *Shewanella oneidensis* MR-1^T^ ^13^) but also in several microbiomes.^11,14^ Direct EET requires physical contact of the microorganisms whereas the indirect EET does not.^15^ Accordingly, the two types of transfer can be assumed to occur preferentially in biofilms or in planktonic cultures, respectively.

The anode compartment of a MET reactor provides a distinct ecological niche mainly characterized by two features. First, the anode serves as TEA for anaerobic respiration via EET. Second, the carbon and energy sources determine the actual carbon and redox reactions at the anode. Whereas the electrode potential can be controlled and is usually set to values between −200 and + 400 mV vs. the standard hydrogen electrode (SHE), the types of carbon and energy sources in WW are highly variable and diverse in terms of chemical composition and concentration. Often, the specific composition cannot be determined and only sum parameters are assessed. The most prominent sum parameter is the chemical oxygen demand (COD), a measure for the oxygen equivalents (and hence the electrons) needed for the oxidation of all WW compounds to CO_2_. Thus, the COD is also the foundation for legal regulations. For instance, a COD ≤ 200 mg L^−1^ is required in Germany for treated WW to be released into the environment.^16^

The reactor microbiome of a MET reactor anode compartment for WW treatment has two major functionalities: first, cleaning WW by oxidation of substrates (removal of COD), second, harvesting the electrons of the oxidation process as electric current by employing the anode as TEA. As electroactive microorganisms are metabolically limited,^14,17^ the challenge of MET engineering is to provide an ecological niche at the anode that fosters COD removal as well as electric current production by building up a well-balanced food web.

For the translation into application, a better understanding of anode colonization and the development of the microbiome under real WW conditions is required. This includes, for instance, understanding the relationship between microbiome composition and functionality in terms of COD removal, current density (*j*), and electron efficiency (Coulombic efficiency, CE). Only very little is known of the ecological principles for the formation and temporal development of microbiomes in MET. Therefore, this study aims to shed light on the structure–function relationship of microbial anodes from initial colonization based on domestic WW to its stable treatment. To distinguish and understand the impact of the different trophic levels on COD removal and current production, WW of different complexity ranging from organic acids to real domestic WW was studied in eleven 500 mL reactors, run in parallel and inoculated identically. Electrochemical performance was continuously monitored and WW-related parameters as well as the microbiome composition of all anodic biofilms and planktonic phases were analyzed for each batch. This approach allowed us to determine the patterns of initial electrode colonization, the development of complex trophic networks, as well as of the key players for COD removal and electric current generation, and to derive general recommendations on the design and steering of anode microbiomes in WWTP.

## Results

### Reactor performance

**Fig. 1:**
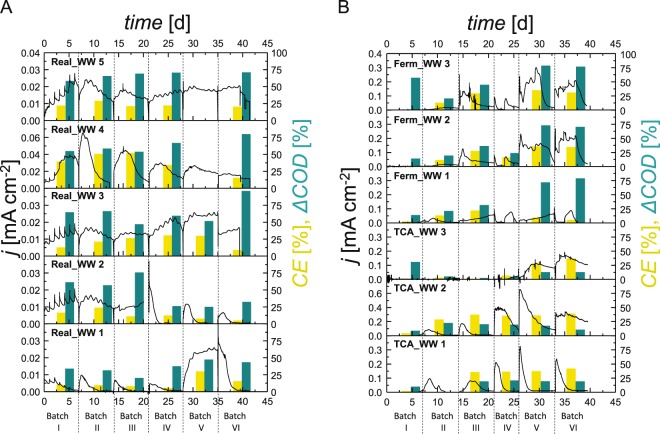
Course of current density (*j*) and characteristic process parameters (Coulombic efficiency (CE, yellow) and COD removal (Δ*COD*, turquoise)) in real wastewater reactors Real_WW 1–5 (**a**) and defined wastewater reactors TCA_WW 1–3 and Ferm_WW 1–3 (**b**). The current density (black line) was continuously monitored for all reactors over the course of the experiment, while the other parameters (colored bars) were determined per batch (detailed values in Supplementary Table 1). Note the different scaling of axis for **a** and **b** to allow the visualization of differences between reactors. The Real_WW 1–5 reactors were run with real domestic wastewater as the only source of carbon and microorganisms. The TCA_WW 1–3 and Ferm_WW 1–3 reactors were inoculated with 5% real domestic WW in the first batch and run all batches with 0.6 g L^−1^ COD equivalents of propionate, butyrate and acetate (TCA_WW 1–3) or sucrose, and propionate, butyrate, and acetate (Ferm_WW 1–3). After each batch, the reactor liquid was completely replenished by fresh real domestic WW, respectively, defined as wastewater

The Real_WW reactors were started without prior enrichment of electroactive biofilms with real domestic WW as the only source of inoculum and substrate. The WW was collected from a local WWTP on average characterized by the following parameters: COD 473 ± 74 mg L^−1^, pH 8.0 ± 0.1, total nitrogen 89 ± 6 mg L^−1^, ammonium 73 ± 27 mg L^−1^, sulfide 0.9 ± 0.4 mg L^−1^, and total organic carbon (TOC) 163 ± 17 mg L^−1^.^18^ All Real_WW reactors started current production already in batch I (Fig. 1) with a maximum current density of 0.04 mA cm^−2^ in Real_WW reactor 4 at day 5. Small cyclic variations in the current density are due to diurnal temperature changes. After each batch, the treated WW was completely removed from the reactor; the reactor was then refilled with fresh deaerated real WW and electrochemically incubated for another week. Cells attached to the electrodes and the glass walls of the reactor vessel remained within the reactor during the exchange. In general, the treated WW appeared more transparent then the fresh WW, but the formation of bigger flocks was observed.

Considering all six batches, the Real_WW reactors showed a similar performance regarding treatment efficiency (ΔCOD = 55 ± 18%) and electrochemical performance (*q* = 351 ± 211 C, CE = 21 ± 12%), with individual exceptions especially for Real_WW reactors 1 and 2. Real_WW reactor 1 showed the lowest performance during the batches I–IV, but then it suddenly increased in current density during batch V. This increase was also reflected in the COD removal efficiency that increased from an average of 30 ± 6% during batch I–IV to 47% and 43% in batches V and VI, respectively. In contrast, Real_WW reactor 2 showed a performance in line with reactors 1–3 over the first three batches (*q* = 316 ± 57 C, ΔCOD = 65 ± 8%) and then declined significantly in batches IV–VI (*q* = 67 ± 17 C, ΔCOD = 28 ± 3%). Nevertheless, a successful COD removal, being the main measure for WW treatment, and current generation was found in all reactors (details in Fig. 1, Supplementary Table 1), and similar characteristics of the treated WW were determined with pH 7.1 ± 0.3, total nitrogen 86 ± 9 g L^−1^, ammonium 14 ± 2 g L^−1^, and TOC 107 ± 32 mg L^−1^. Only the sulfide concentration differed ranging from below 0.5 g L^−1^ in Real_WW reactors 1 and 2 to above 20 g L^−1^ for the other reactors.

The reactors with defined WW (Ferm_WW and TCA_WW) were started with 5% real WW as inoculum in batch I. No prior enrichments and no additional inoculation were performed in consecutive batches. In contrast to the Real_WW, all defined WW reactors showed increased performance over time. While maximum current densities in the first batch only reached 0.03 mA cm^−2^ (TCA_WW reactors 2 and 3) and the average charge was *q* = 40 ± 15 C, these values increased significantly in the following batches with the highest values in batches V and VI. Here, the maximum current densities were one order of magnitude higher than that for Real_WW (e.g., *j* = 0.30 mA cm^−2^ batch V, TCA_WW reactor 3 and *j* = 0.03 mA cm^−2^ batch V, Real_WW reactor 4). The accumulated charge was about three times higher for the defined WW with Ferm_WW 1–3 (*q* = 2178 ± 1134 C, batch VI) for WW based on organic acids and sugar compared with TCA_WW 1–3 (*q* = 773 ± 224 C, batch VI) exploiting WW based on organic acids only. While the charge production (*q* = 803 ± 369 C for TCA_WW and *q* = 1731 ± 1389 C for Ferm_WW) and COD removal (Δ*COD* = 15 ± 5% for TCA_WW, Δ*COD* = 68 ± 5% for Ferm_WW) differed significantly between the two types of defined WW (*p* = 0.03 and *p* = 0.00, student’s *t* test considering batches IV–VI), this difference was not found for the CE (*p* = 0.10) being 33 ± 10% and 24 ± 12% (batches IV–VI).

While the individual degradation pathways for the complex substrate composition in domestic WW are difficult to determine, the fate of individual substrates was studied by using the defined WW. The concentrations of sucrose (only in Ferm_WW) and acetate, propionate, and butyrate (in TCA_WW and Ferm_WW) were determined after each batch (Table 1). In general, the substrates were not only degraded bioelectrochemically, as the maximum CE was only 42%. Ferm_WW sucrose was always completely removed. The main share of the primary degradation was most likely performed by planktonic cells as only in Ferm_WW 1–3, the planktonic phase was turbid and showed a visible biomass increase. As a result of the sucrose conversion, an accumulation of acetate and propionate was found in batches I–IV. Nevertheless, the partial degradation of the organic acids resulted in an average COD removal over all batches of 15 ± 7% (TCA_WW 1–3) and 46 ± 25% (Ferm_WW 1–3). Further, a reddish biofilm formation at the anodes was observed in all reactors.

**Table 1:** Average substrate conversion in the defined wastewater reactors

	Sucrose	Acetate	Propionate	Butyrate
*TCA_WW reactors 1–3*				
Batch I	−	−0.1 ± 0.5	−0.7 ± 0.4	−0.7 ± 0.4
Batch II	−	−1.6 ± 1.3	−0.3 ± 0.2	−0.2 ± 0.0
Batch III	−	−1.4 ± 1.2	−0.5 ± 0.3	−0.1 ± 0.1
Batch IV	−	−1.7 ± 1.3	−0.4 ± 0.2	−0.2 ± 0.1
Batch V	−	−2.0 ± 0.5	−0.5 ± 0.2	−0.2 ± 0.1
Batch VI	−	−1.7 ± 0,4	−0.5 ± 0.3	−0.0 ± 0.2
*Ferm_WW reactors 1–3*				
Batch I	−2.0 ± 0.0	4.1 ± 3.8	1.7 ± 0.6	0.1 ± 0.0
Batch II	−2.0 ± 0.0	6.4 ± 0.4	1.1 ± 0.2	0.1 ± 0.0
Batch III	−2.0 ± 0.0	3.6 ± 1.0	1.3 ± 0.4	−0.0 ± 0.0
Batch IV	−2.0 ± 0.0	6.0	0.9	0.2
Batch V	−2.0 ± 0.0	−1.0 ± 0.0	0.5 ± 0.2	0.0 ± 0.0
Batch VI	−2.0 ± 0.0	−1.0 ± 0.0	0.5 ± 0.2	−0.1 ± 0.1

Negative values represent a degradation compared with the start concentration. Positive values indicate that the concentration of the compound after a batch was higher than the initial concentration of the batch, e.g., as a result of the fermentation of sucrose to organic acids. The values give average ± standard deviation in mM (*n* = 3), except for Ferm_WW batch IV (*n* = 1)

### Bacterial community analysis

All reactors were started by using the identical real domestic WW inoculum at the identical point of time. Clear differences in the electrochemical performance were observed for the different reactor setups containing either Real_WW or a TCA_WW and Ferm_WW. Yet, also differences in the performance of the parallel-run reactors were evident (see the section above). These differences are most likely the result of stochastic biological processes,^19,20^ as shown previously also for the colonization of anodes.^21^ Therefore, the primary colonization of the anodes from the domestic WW inoculum was determined and followed in its development over time.

The real WW had a stable community composition over six batches (60–70% Proteobacteria, 11–13% Firmicutes, and 12–19% Bacteroidetes) although it was freshly collected every week (complete data set with relative abundance data of all genera in all samples is provided as Supplementary Data 1). It was further dominated by the genus *Arcobacter* (*Epsilonproteobacteria*) to which 47 ± 3% of all sequences were assigned. *Arcobacter* has been found in numerous other environmental samples, i.e., estuarine sediment, marine water,^22,23^ but its functional relevance and the reason for its dominance in these WW reactors are not known. The community composition of the treated WW (planktonic phase) in the Real_WW reactors 3–5 is highly similar to the original WW and only little variation was found over the different batches. Real_WW reactors 1 and 2 possess a higher variability in the community composition of the treated WW but show similar trends. The contribution of *Arcobacter* varies in the treated real WW samples between 1% and 48%.

**Fig. 2:**
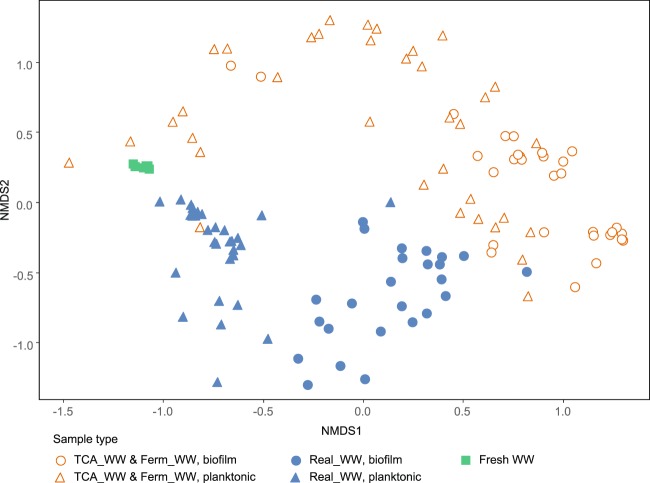
Bacterial community composition in all samples based on nonmetric multidimensional scaling. The color code of the symbols represents the different reactors running with real (Real_WW) or defined wastewater (TCA_WW and Ferm_WW) as well as the sample origin being the fresh domestic wastewater, biofilm, or planktonic phase. A more detailed assignment of the individual reactors can be found in Supplementary Fig. 1

**Fig. 3:**
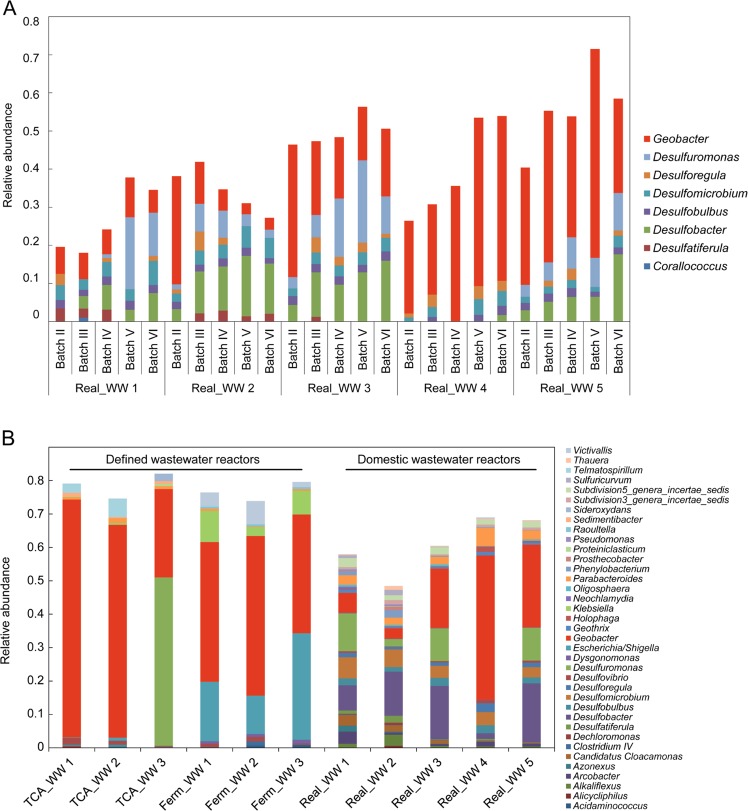
Bacterial community composition. **a** Relative abundance of *Deltaproteobacteria* in the anode biofilms in the Real_WW reactors 1–5 over time. The biofilms formed at the electrode during batch I were not sufficiently dense for sampling and therefore had to be excluded. The relative abundance refers to all sequences in each sample but only genera of *Deltaproteobacteria* with a contribution of more than 1% are displayed. **b** Community composition of the anode biofilms after batch VI, including all genera with an abundance of at least 1%. The defined wastewater reactors TCA_WW 1–3 and Ferm_WW 1–3 were inoculated with 5% domestic wastewater for batch I. In contrast, Real_WW reactors 1–5 received fresh real WW in each batch as the only source of carbon and repeated inoculum

The biofilm samples of the Real_WW reactors 1–5 clearly differ from the inoculum and the planktonic phase (Fig. 2). This shows a specific enrichment of a functional bacterial community by growth on the anode surface rather than a random attachment of bacterial cells. The biofilm samples show a similar composition on the genus level, but differ in the individual abundances with similar trends over time. Especially, the enrichment of *Deltaproteobacteria* became obvious in all biofilms starting with 34 ± 10% in batch II and increasing to 45 ± 12% in batch VI. Eight different genera were assigned to the *Deltaproteobacteria* (Fig. 3) with *Geobacter* having the highest contribution over all samples with a relative abundance of 22 ± 14%. Especially in the Real_WW reactors 4 and 5, *Geobacter* clearly dominated the bacterial community with 34 ± 9% (Real_WW reactor 4) and 36 ± 10% (Real_WW reactor 5). The second most abundant *Deltaproteobacteria* are *Desulfobacter* (9 ± 5%) and *Desulfuromonas* (8 ± 6%). These two genera are known for sulfate and sulfur reduction^24^ and *Desulfuromonas* even has an electroactive representative.^17^

The anode biofilms of the Ferm_WW reactors 1–3 and TCA_WW reactors 1–3 also clearly differed from the inoculum and clustered closer to the anode biofilms of the Real_WW reactors (Fig. 2). The anode biofilms of the defined WW reactors also show a high enrichment of *Deltaproteobacteria* with 45 ± 22%. *Geobacter* seems to be an efficient first colonizer with 25 ± 12% in batch II. Nevertheless, also the enrichment of other *Deltaproteobacteria* (*Desulfuromonas* up to 50% in TCA_WW reactor 3, *Desulfobulbus* up to 1%) followed in the subsequent batches, although the inoculation was only performed in batch I. This indicates that the other species also attached to the anode during batch I, but did not increase their abundance as efficient as *Geobacter* which is well-known for its dominance in electroactive biofilms in similar experiments based on artificial WW.^25–27^ Besides *Deltaproteobacteria*, also other bacteria contributed to the anode biofilms with a clear difference between TCA_WW and Ferm_WW and for both in comparison with Real_WW (Fig. 3b). The TCA_WW reactors were clearly dominated by *Geobacter* after batch VI, except TCA_WW 3 showing also a high contribution of *Desulfuromonas*.

While the acetate oxidation and related current generation is very likely performed by *Geobacter* as the key player, it is an open question how the oxidation of propionate and butyrate is realized. *Geobacter anodireducens* has the physiological capacity for propionate utilization if acetate is also present.^28^ In the Ferm_WW reactors, the genus *Escherichia*/*Shigella* had a significant contribution with up to 20 ± 9% in batch VI. The abundance of this genus was below 1% in all other reactors indicating its specific role for the sugar fermentation. In the Real_WW reactors, the substrate range is much broader than that in the defined WW reactors. This is also reflected by the community composition of the anodic biofilms showing a high diversity and no dominance of single genera (Fig. 3).

## Discussion

Generally, the planktonic phase of the reactors treating real WW (Real_WW 1–5) was more similar to the inoculum than to the anodic biofilms. This indicates that with the applied anode surface area to reactor volume ratio, the anaerobic electrochemical incubation had only little impact on the composition of the planktonic community. The anodes were immediately colonized within batch I as shown by the current production that sustained for the subsequent batches with anode biofilm communities being clearly different from the initial WW inoculum. These results clearly show that the overall performance in terms of COD degradation and electrochemical activity is mainly governed by the anode biofilms. Nevertheless, alternative reactions like sulfur reduction (see discussion below) or methanogenesis can occur and contribute to COD degradation, thus explaining CE values below 40%.

**Fig. 4:**
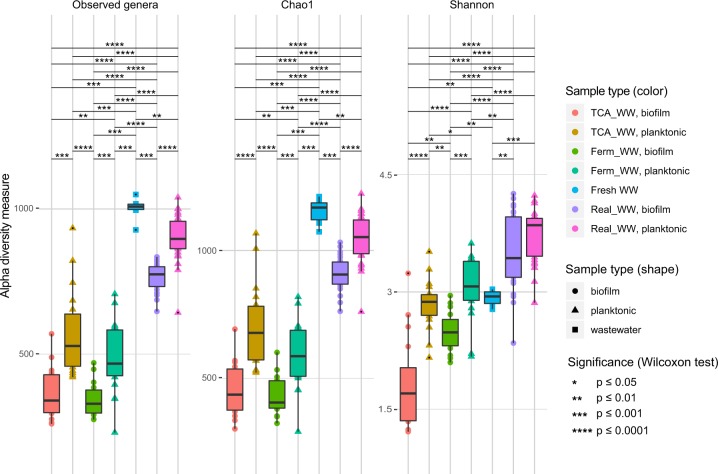
α-Diversity indices in all samples based on the diversity measures Observed genera, Chao1, and Shannon. The color code of the symbols represents the different reactors running with real (Real_WW) or defined wastewater (TCA_WW and Ferm_WW). The shape of the symbols is referring to the sample origin, the fresh domestic wastewater (square), biofilm (circle), or planktonic phase (triangle). The calculation of *p* values was performed with the R packages vegan, iNext,^27^ and RDPutils^23^

For elucidating the structure–function relationships, the correlation of anode communities and the overall performance of the reactors were analyzed. While the defined WW reactors (TCA_WW and Ferm_WW) increased their performance from batch I to VI, indicating an adaptation with functional specialization of the microbiome, this performance increase was not observed in the Real_WW reactors. This adaptation with functional specialization in bacterial community composition is mirrored by the diversity of the anode biofilm communities in batch VI (Fig. 3b) but can also be clearly seen when considering all batches. The significantly highest values of the α-diversity indices (Observed genera and Chao1) within the different sample types are found in the fresh WW samples (1002 ± 40, 1153 ± 52, Fig. 4, the complete data set with all values is provided as Supplementary Data 1). Being derived from a WWTP every week, the bacterial community in these samples had probably experienced high variations regarding environmental parameters and daily fluctuations (e.g., COD composition of inflow, weather, etc.) resulting in the variable composition of the bacterial community. At the same time, higher Shannon values in the biofilm (3.5 ± 0.48) and planktonic (3.7 ± 0.33) samples of the Real_WW reactors in comparison with the Fresh_WW samples (2.9 ± 0.10, Fig. 4) suggest a more even distribution of the identified bacterial genera in the reactors. Hence, the reactors provide a relatively stable environment and hence a well-defined ecological niche. While in comparison with the Fresh_WW samples, the planktonic samples of Real_WW show only slightly significantly lower values of the α-diversity indices of 902 ± 78 (Observed genera) and 1046 ± 94 (Chao1); the high specialization of the anode biofilm communities to the ecological niche of the anode becomes evident from the highly significantly lower α-diversity values of 765 ± 49 (Observed genera) and 910 ± 66 (Chao1), respectively. Furthermore, as the WW is the only source of carbon and electrons in the Real_WW reactors, the diversity still has to be relatively high to allow utilization of different and even complex carbon sources. While the electroactive members of the anode biofilm are most likely metabolically limited, as most electroactive species can only utilize simple sugars and small organic acids,^14,17^ the degradation of more complex compounds from the WW has to be performed by other, probably non-electroactive, members of the community.

In contrast, feeding the reactors with defined WW allows a higher degree of adaptation and specialization in the respective biofilms. This specialization goes along with higher functional performance (see Fig. 1) and significantly lower α-diversity values (TCA_WW: 369 ± 92 Observed genera, overall 458 ± 107 genera according to the Chao1 estimator, Shannon diversity 1.8 ± 0.62; Ferm_WW: 343 ± 60 Observed genera, 430 ± 78 Chao1, and 2.5 ± 0.26 Shannon) compared with the Real_WW biofilms (765 ± 49 Observed genera, 910 ± 66 Chao1, and 3.5 ± 0.48 Shannon). The specialization also takes place in the planktonic communities of the defined WW reactors (TCA_WW: 567 ± 147 Observed genera, 704 ± 159 Chao1, and 2.8 ± 0.32 Shannon; Ferm_WW: 483 ± 121 Observed genera, 593 ± 130 Chao1, and 3.0 ± 0.40 Shannon) with α-diversity values being significantly lower than the Real_WW samples (902 ± 78 Observed genera, 1046 ± 94 Chao1, and 3.7 ± 0.33 Shannon, Fig. 4). While this specialization is advantageous for the electrochemical performance, it can be a limitation when changes in the WW composition occur and a higher flexibility is required for COD removal and current production (see discussion below).

By comparing the functional performance of all reactors with their bacterial community composition, the key role for the genus *Geobacter* is obvious (Fig. 3) and in accordance with previous observations, e.g., refs ^25,29^.

By considering all reactors, only the abundance of *Geobacter* shows a correlation with the CE (*r* = 0.56) that is higher than 0.5 (all data given in Table 2). In contrast, the abundance of *Geobacter* does not significantly correlate to the current production and COD removal although positive trends between *Geobacter* abundance and performance parameters can be seen for the individual reactors. For the combined data set of all reactors, the abundance of several other genera possesses a correlation higher than 0.5 (Table 2). When considering only the data of the biofilms from the Real_WW reactors, the correlation between CE and *Geobacter* is less pronounced and also other taxa contribute to the same (Supplementary Table 2). This supports the specific role of *Geobacter* for the efficiency in terms of current production from oxidation of small organic acids. The degradation of more complex organic compounds depends on other microbial community members that vary for the respective reactors and depend on the provided substrates as well as stochastic events as, e.g., the difference in the presence of low abundant microorganisms during inoculation and colonization.

**Table 2:** Correlation analysis of all reactors: positive and negative correlations of the relative abundance of microbial genera with reactor performance parameters considering the complete data set with all reactors (Real_WW, Ferm_WW, and TCA_WW)

	*q*	CE	COD removal
*Acidaminococcus*	0.79		
*Akkermansia*			0.50
*Anaerovorax*			0.59
*Bacteroides*	0.55		
*Clostridium*_IV	0.53		
*Desulfobulbus*	−0.51		
*Dialister*			0.51
*Escherichia/Shigella*	0.58		
*Gemmiger*			0.58
*Geobacter*		0.56	
*Geothrix*			0.52
*Phascolarctobacterium*	0.72		

Only values above 0.5 (positive correlation) and below −0.5 (negative correlation) are shown

**Fig. 5:**
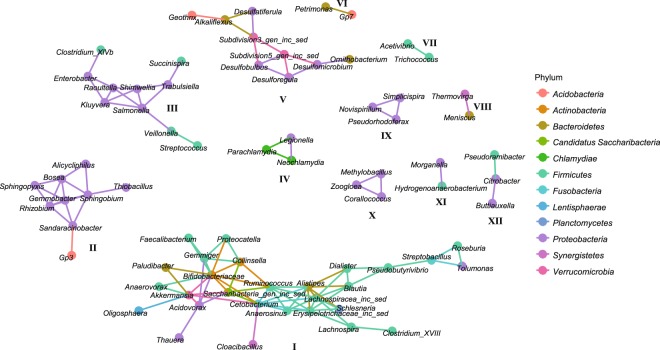
Microbiome interaction network. Interestingly, as a result of this analysis, *Geobacter* is not included; thus, it is not connected to the other genera. This supports the hypothesis that *Geobacter* occupies a specific ecological niche within the anode biofilm independent from general COD degradation and other microorganisms. The biggest cluster (I) has the highest importance for the functional performance of the reactors followed by cluster V, while the members of cluster II show negative correlations to reactor performance parameters and are probably responsible for alternative COD degradation pathways being independent of anode respiration

When now analyzing the potential interactions among the bacteria based on microbiome network analysis (Fig. 5), *Geobacter* is very surprisingly absent from the network covering 74 out of 231 genera. The most important clusters in terms of performance are clusters I and V. Most members of these clusters show a positive correlation to the performance parameters (Supplementary Table 2) and in addition cluster V relates positively to sulfide production being in accordance with the higher sulfide concentration in the Real_WW reactors 3–5. While the increase in sulfide due to sulfur reduction feels counterintuitive for the successful anode respiration, the respective taxa might have the capacity to perform both anode respiration as well as sulfur reduction. In contrast, the members in cluster II including, e.g., *Thiobacillus* and *Rhizobium*, show mainly negative correlations to the bioelectrochemical WW degradation in terms of CE and *j*. Hence, they are probably involved in COD degradation pathways (positive correlation for COD) independent from the anode. This could explain the lower performance of Real_WW reactor 1 as here these genera had higher contributions to the anodic biofilm community than those in the other Real_WW reactors. Other clusters like clusters III and IV that also contain potential disease-associated genera (*Enterobacter*, *Salmonella*, and *Legionella*) seem independent from the reactor performance. Yet, they show a clear co-occurrence that is, e.g., related to the presence of sucrose in the Ferm_WW reactors 1–3 for cluster III.

While being present in all biofilms, the absence of the genus *Geobacter* from the network means that it does not show a distinct correlation with one or a few other genera over all anode biofilms. This supports previous observations and the specific ecological role, as well as the outstanding functionality of *Geobacter* in anode biofilms. *Geobacter* is the fastest and most competitive primary colonizer of the provided ecological niche being an anode surface serving as TEA. However, *Geobacter* is not able to degrade complex substrates as those usually found in WW. Thus, other members being part of the planktonic community (e.g., those in cluster I) or the biofilm (e.g., those in cluster V) are required to develop a trophic network allowing COD degradation as well as current production. Thus, there is no cluster including typically known electroactive microorganisms.^17^ Therefore, the question persists, if *Geobacter* as the successful first colonizer remains also the only electroactive taxon in these biofilms over time, or if other taxa either using direct or mediated EET also contribute to current generation. Altogether, the microbiome network analysis leads to the conclusion that it is not an individual genus that is governing the reactor performance. Rather, the diversity and complexity of all members—and their division of labor—within the trophic network is of functional importance with *Geobacter* driving the reactor performance regarding CE.

The initial colonization of an anode surface is very likely to be performed by *Geobacter anodireducens* together with less- abundant species. Over the course of WW treatment, these species can increase their abundance and adapt the biofilm composition in a flexible way toward the specific substrates provided as well as other important ecological parameters like temperature, pH, the presence of alternative electron acceptors, etc.^30^ While pre-enrichment of *Geobacter* on anodes is often performed in laboratory reactors (e.g., refs ^31,32^), this aspect is usually not considered for the other microbial community members, which makes their contribution to electrochemical performance and COD removal rather stochastic.

What does this now mean for the implementation of MET for WW treatment? Generally, reactor microbiomes that are less diverse and more stable in terms of taxon composition can provide the highest performance under fixed conditions (i.e., highly reproducible conditions and WW composition, e.g., specific industrial WW). This is also the case for microbial anodes that are usually achieved by pre-enrichment from real WW using acetate as the only substrate. This is certainly highly suitable for fundamental studies and can also be adopted for technical implementation in case the targeted WW contains acetate or a COD that can be easily converted therein. The secondary colonization by the microorganisms providing the acetate from COD, will then be realized by the natural community of the WW. But one has to keep in mind that such a high specialization of a microbial biofilm is very sensitive to any change of the growth conditions which can result in significant functional losses up to complete biofilm detachment (see e.g., ref. ^31^). Most WW is less stable in its composition and environmental conditions are usually fluctuating. In these cases, colonization of the pre-enriched anode within the final habitat will result in a higher variability regarding anode biofilm composition. Most likely, functional anodic biofilms will be formed, but there is still the chance that the primary colonization, e.g., by *Geobacter anodireducens*, is not the optimum for the WW to be treated. Here, a specific recommendation is case dependent. One option would be to pre-colonize the anode with a very diverse microbial inoculum and use of a highly diverse substrate (being representative of the average WW to be treated, see e.g., ref. ^33^). Once a diverse anode biofilm is established, it can be transferred to the site of application. Also here, realistic abiotic conditions should be applied for the pre-enrichment to allow a transfer to practice. In conclusion, the specific composition and variability of the WW to be treated has to be accounted for optimal process development under consideration of general ecological principles.^30^ Therefore, we assume WW of more constant composition, e.g., from food industries, being more likely to be treated using MET than municipal WW.

## Methods

### Chemicals, potential reference, and WWs

All chemicals were of analytical or biochemical grade. All potentials provided refer to the SHE by conversion from Ag/AgCl (sat. KCl, +197 mV vs. SHE).

Three types of WW were used in order to address three different metabolic capacities and hence trophic levels that are relevant for successful COD degradation: two defined WWs allowing either only tricarboxylic acid (TCA) cycle (TCA_WW) or allowing TCA and glycolysis (Ferm_WW), and real domestic WW (Real_WW). For better comparison, the charge, coulombic efficiency, and the removal of organic compounds (in all reactors monitored in terms of COD equivalents) are given in Supplementary Table 1. In addition, the chemical composition was investigated in detail for the reactors run with TCA_WW and Ferm_WW using HPLC.

TCA_WW and Ferm_WW were based on a carbonate-buffered mineral medium^29^ that contained 30 mM of the carbon source equaling a COD of 0.6 g L^−1^ in both cases. TCA_WW contained 3.75 mM sodium propionate, 3.75 mM sodium butyrate, and 2 mM sodium acetate. Ferm_WW contained 2 mM sucrose, 1 mM sodium propionate, 1 mM sodium butyrate, and 1 mM sodium acetate. The Real_WW had an average COD of 473 ± 74 mg L^−1^.

Fresh WW was obtained weekly from the primary clarifier (after passing mechanical filtration) of the municipal WWTP (*Abwasserzweckverband für die Reinhaltung der Parthe, Am Klärwerk, 04451 Borsdorf)*, Germany. If not used immediately, it was stored at 4 °C for a maximum of 24 h. Prior to usage, the solids in the WW were resuspended by shaking. The fresh WW served as the sole growth medium and inoculum for the Real_WW reactors and was used as inoculum for TCA_WW and Ferm_WW reactors by adding 25 mL in a 500-mL reactor at the beginning of the experiments (i.e., batch I, see also below).

### Experimental setup

MET reactors were based on tailor-made glass reactors^34^ and shown in Supplementary Fig. 2. Working electrodes (WE) and counter electrodes were made from graphite rods and plates (CP-2200 quality, CP-Handels GmbH, Wachtberg, Germany). The WE and counter electrodes had 47.6- and 15 cm^2^ geometric surface area, respectively, and were separated by a membrane (fumasep® FKE, Fumatech GmbH, Bissingen, Germany). WE chambers contained 500 mL of WW leading to an anodic surface-area-to-volume ratio of 95.2 cm^2^ L^−1^ at the beginning and 76 cm^2^ L^−1^ at the end of the experiment, i.e., after six batches of operation (due to sampling, see below). Counter electrode chambers were filled with 10 mL of a phosphate buffer (1.8 g L^−1^ Na_2_HPO_4_, 0.223 g L^−1^ NaH_2_PO_4_, and 8.5 g L^−1^ NaCl, pH 7.2). Reference electrodes (Ag/AgCl sat. KCl, SE 11, Meinsberg Sensortechnik GmbH, Germany) were introduced in the WE chamber. WE were poised at +397 mV vs. SHE with recording of current every 10 min by using a multipotentiostat (MPG-2, Bio-Logic SAS, Claix, France). Note that the potentiostatic operation assured that differences in the electrolytic conductivity did not have an impact on the potential of the WE.

Fresh WW was introduced in the WE chambers and purged with nitrogen for 25 min. The experiments were conducted at room temperature and stirring at 150 rpm by using magnetic stirrers. Three independent replicate reactors were run for each TCA_WW and Ferm_WW and five independent replicate reactors for Real_WW. All reactors were started and run in parallel.

The experiment was run over six batches, which were labeled accordingly as batches I–VI, for a duration of 7 days per batch. The time for each batch was set constant to 7 days in order to synchronize the development of the reactors without facing starvation periods. After each batch, the WW of all reactors was completely exchanged and liquid samples of the treated WW (planktonic phase) and biofilm samples of the WE were collected. The WE was sampled by cutting a defined piece of the plate (1 cm length and hence 2.4 cm² surface area, Supplementary Fig. 2B). Subsequently, fresh WW was introduced in the anode chambers and purged with nitrogen for 25 min. The TCA_WW and Ferm_WW reactors received the respective sterile WW without any inoculate in batches II–VI. Each batch the cathode chamber was also refilled with new buffer solution. Chemical analysis (COD, TOC, sulfate, total nitrogen, nitrate, ammonium, phosphate, conductivity, pH of the fresh and treated real_WW, HPLC analysis of sucrose, acetate, propionate, and *n*-butyrate for TCA_WW and Ferm_WW) was performed using standard methods (details are given in Supplementary methods).

### DNA extraction

The cut piece of the WE was stored at −20 °C until DNA extraction. In total, 3 mL of treated WW were centrifuged for 10 min with 14,000*g* at 4 °C. The supernatant was removed and the pellet stored at −20 °C until DNA extraction. DNA was extracted using the NucleoSpin^(R)^ Tissue Kit (Macherey Nagel, Düren, Germany). The WW pellet was redissolved in the first extraction buffer containing T1 + Proteinase K. The same buffer was also added to the WE piece. All samples were incubated at 56 °C for 2 h and then further processed according to the manufacturer’s recommendation.

### Amplicon preparation, next-generation sequencing, and amplicon analysis pipeline

High-throughput sequencing of the 16S rRNA gene (V3 region) was applied to determine bacterial community composition of the fresh domestic WW, the treated WW, and the anodic biofilm samples. A preamplification of the V3 region of the 16S rRNA gene (primer pair 341f–515r) was followed by amplicon preparation as described by Bartram et al.^35^ Amplicons were sequenced on the Illumina NextSeq550 platform (San Diego, CA, USA) in 150-bp pair-end mode and generated a total of 334,203,639 bacterial sequences which were quality-checked by the FastQC program version 0.10.1 (Simon Andrews; http://www.bioinformatics.babraham.ac.uk/projects/fastqc/). Due to quality loss at the ends of forward and reverse reads, those were trimmed to a length of 130 bp. A JAVA program *DimerFilter* based on FastQC removed potential primer dimers from the raw sequence data. Forward and the reverse reads were joined by fastq-join^36^ using a 20% mismatch and a minimum overlap of 6 bp. Result files were converted to FASTA and checked for chimeras with *Uchime* (Usearch 5.2.32^37^) against the gold database provided by ChimeraSlayer (http://drive5.com/otupipe/gold.tz). Finally, RDP classifier version 2.10.1^38,39^ was applied for taxonomic-dependent analysis employing a confidence value of 0.5, as recommended for short-read amplicon data.

### Data analysis and statistics

The current density (*j*) is provided per geometric surface area and the Coulombic efficiency (CE) was calculated based on the COD equivalents (see also Supplementary Table 3). The experimental data were analyzed with Excel and Origin and the phylogenetic data with R using the package phyloseq.^40^ The presented data give the average ± standard deviation of three independent biological replicates for the TCA_WW and Ferm_WW reactors and of five independent biological replicates of the Real_WW reactors. Where appropriate, the individual data of each reactor are shown. Correlation analysis was based on Spearman’s rank correlation coefficient using the R package vegan.^41^ The calculation of α-diversity values and the rarefaction curves (Supplementary Fig. 3) was performed with the R packages vegan, iNext,^42^ and RDPutils.^38^

### Reporting summary

Further information on research design is available in the Nature Research Reporting Summary linked to this article.

## Supplementary information

Supplementary Material

Supplementary Data 1

Reporting Summary

## Data Availability

The data that support the findings of this study are available from the corresponding author upon reasonable request.
