# Supplementary material for: Trophic networks improve the performance of microbial anodes treating wastewater
Source: NPJ Biofilms Microbiomes. 2019 Sep 27;5:27. doi: 10.1038/s41522-019-0100-y (PMC6764952; doi:10.1038/s41522-019-0100-y)
Supplement: Supplementary file 1 — Supplementary Material [file 41522_2019_100_MOESM1_ESM.pdf]

Supplementary Information

Trophic networks improve the performance of microbial anodes treating wastewater

Christin Koch[a,*,#], Katharina J. Huber[b], Boyke Bunk[b], Jörg Overmann[b,c], Falk Harnisch[a,*]

[a] Helmholtz-Centre for Environmental Research, Department of Environmental Microbiology Permoserstraße 15, 04318 Leipzig, Germany
[b] Leibniz Institute DSMZ – German Collection of Microorganisms and Cell Cultures, Inhoffenstraße 7B, 38124 Braunschweig, Germany
[c] Department of Life Sciences, Braunschweig University of Technology, Germany
# Current affiliation: Global Innovation Cosmetic Ingredients, Symrise AG, Mühlenfeldstraße 1, 37603 Holzminden, Germany,

Supplementary Material for Results and Discussion sections

Supplementary Table 1	Reactor performance	Page 2
Supplementary Table 2	Correlation performance domestic wastewater reactors	Page 3
Supplementary Figure 1	Microbial community composition in all samples based on non-metric multidimensional scaling	Page 5
Supplementary Data 1	Complete data set of relative abundance data of all genera and diversity indices	Additional file

Supplementary Material for Methods section

Supplementary Figure 2	Reactor setup	Page 7
Supplementary methods	Chemical analysis	Page 8
Supplementary Table 3	Chemical reactions of the anodic half cell	Page 9
Supplementary Figure 3	Rarefaction curves	Page 10

Supplementary Table 1: Reactor performance. Characteristic process parameters of domestic wastewater (Real_WW) and defined wastewater reactors (TCA_WW, Ferm_WW).

		Domestic wastewater reactors					Defined wastewater reactors					
		Real_WW					TCA_WW			Ferm_WW		
		1	2	3	4	5	1	2	3	1	2	3
Charge<br>*q* in C	Batch I	103	338	275	707	428	29	58	59	47	23	25
	Batch II	75	373	386	796	514	780	960	23	368	278	317
	Batch III	40	238	348	765	405	963	1109	3	893	1268	1646
	Batch IV	46	84	450	549	385	1083	1012	45	501	489	498
	Batch V	514	73	572	524	440	1148	1052	567	889	3287	3385
	Batch VI	163	44	217	306	371	1088	583	648	576	3049	2909
COD removal<br>ΔCOD in %	Batch I	34	61	65	55	59	10	9	31	14	14	57
	Batch II	31	57	67	59	66	n.d.	23	5	21	19	20
	Batch III	20	76	48	54	70	19	21	2	32	36	44
	Batch IV	37	26	60	67	71	21	20	5	n.d.	24	n.d.
	Batch V	47	25	52	n.d.	n.d.	19	18	13	72	73	78
	Batch VI	43	33	96	80	71	19	11	13	79	70	77
Coulombic efficiency<br>*CE* in %	Batch I	9	17	13	39	22	2	5	1	3	1	0
	Batch II	9	24	22	51	29	n.d.	29	3	13	11	12
	Batch III	7	11	27	52	21	36	38	1	22	28	29
	Batch IV	5	13	31	34	22	37	36	7	n.d.	16	n.d.
	Batch V	30	8	30	n.d.	n.d.	37	36	28	10	36	34
	Batch VI	15	5	9	15	20	42	40	37	6	34	30

n.d. not determined

Supplementary Table 2: Correlation analysis of domestic wastewater reactors: Positive and negative correlations of the relative abundance of microbial genera with reactor performance parameters considering only the domestic wastewater reactors Real_WW 1 to 5. Shown are only values above 0.5 (positive correlation) and below -0.5 (negative correlation).

	q	*CE*	*COD removal*
Acetobacterium			0.59
Acidovorax			-0.69
Acinetobacter		0.62	
Aeromonas		0.62	
Alicycliphilus	-0.66	-0.54	-0.74
Alkaliflexus	-0.71	-0.55	-0.74
Arcobacter	0.59	0.71	
Azospira	-0.55		-0.61
Azovibrio			-0.53
Bacteroides	0.73	0.79	
Bifidobacterium	0.63	0.66	
Blautia	0.60	0.72	
Bosea	-0.69	-0.53	-0.79
Catellibacterium			-0.51
Cloacibacillus	0.56	0.62	
Collinsella	0.63	0.62	
Comamonas		0.53	
Dechloromonas	-0.58	-0.50	-0.59
Desulfatiferula	-0.74	-0.57	-0.70
Desulfomicrobium	-0.58	-0.53	
Desulfovibrio	0.55		0.63
Dialister	0.64	0.58	0.52
Dysgonomonas	0.59	0.60	
Faecalibacterium	0.71	0.76	
Gemmiger	0.74	0.69	0.59
Gemmobacter	-0.65		-0.80
Geobacter	0.56		0.70
Geothrix	0.63	0.62	
Gp3	-0.60		-0.67
Gp7	-0.51		-0.51
Microvirga	-0.50		-0.58
Nitrobacter	-0.55		-0.67
Opitutus			-0.54
Paludibacter	0.63	0.72	
Parabacteroides	0.63	0.69	
Pelobacter			-0.65
Petrimonas	-0.63	-0.55	
Phenylobacterium	-0.65	-0.51	-0.79
Prosthecobacter	-0.55		-0.67
Pseudobutyrivibrio	0.60	0.72	
Pseudomonas			-0.68

Pseudoxanthomonas			-0.51
Rhizobium	-0.69	-0.54	-0.78
Rhodobacter	-0.66		-0.86
Rhodoblastus			-0.61
Ruminococcus	0.62	0.73	
Sandaracinobacter	-0.72	-0.57	-0.84
Sphingobium	-0.74	-0.56	-0.85
Sphingomonas			-0.54
Sphingopyxis	-0.51		-0.60
Stenotrophomonas	-0.55		-0.64
Streptobacillus	0.58	0.69	
Subdivision3_genera_incertae_sedis			-0.67
Sulfuricurvum	-0.74	-0.59	-0.77
Sulfurospirillum	0.58	0.67	
Thauera	-0.61		-0.66
Thiobacillus			-0.55
Uruburuella	0.63	0.69	
Victivallis	0.51	0.51	

A

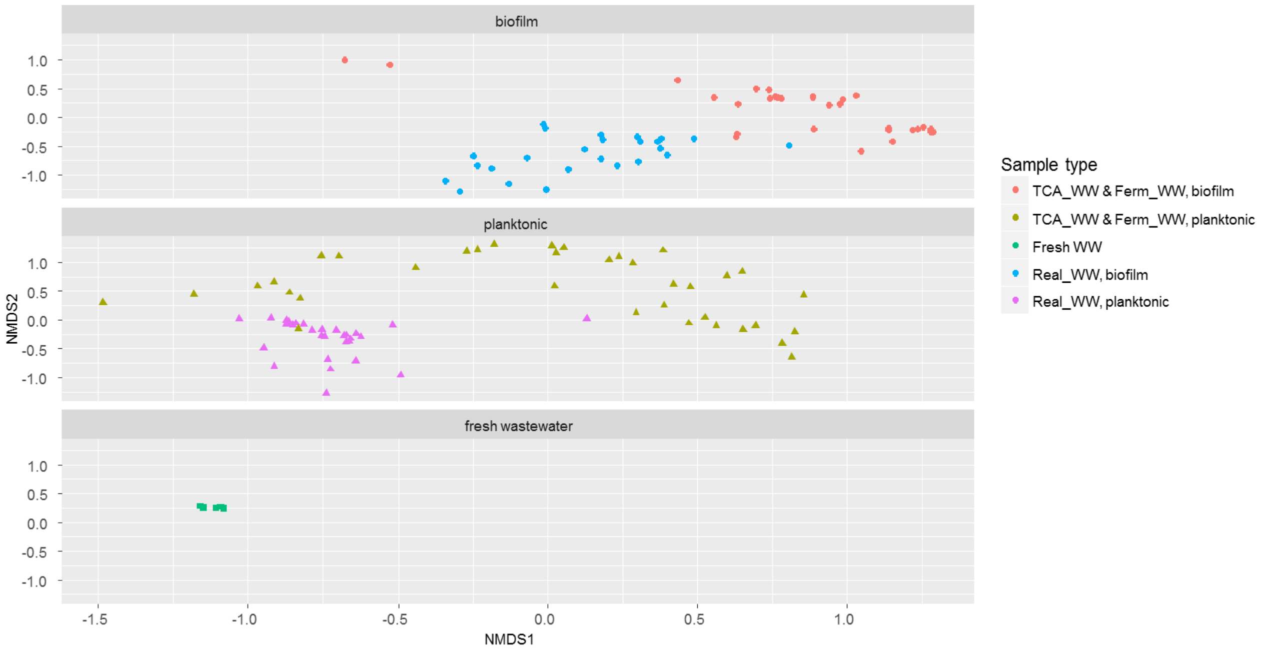
biofilm
planktonic
fresh wastewater
NMDS2
NMDS1
1.0
0.5
0.0
-0.5
-1.0
-1.5
Sample type
TCA_WW & Ferm_WW, biofilm
TCA_WW & Ferm_WW, planktonic
Fresh WW
Real_WW, biofilm
Real_WW, planktonic

B

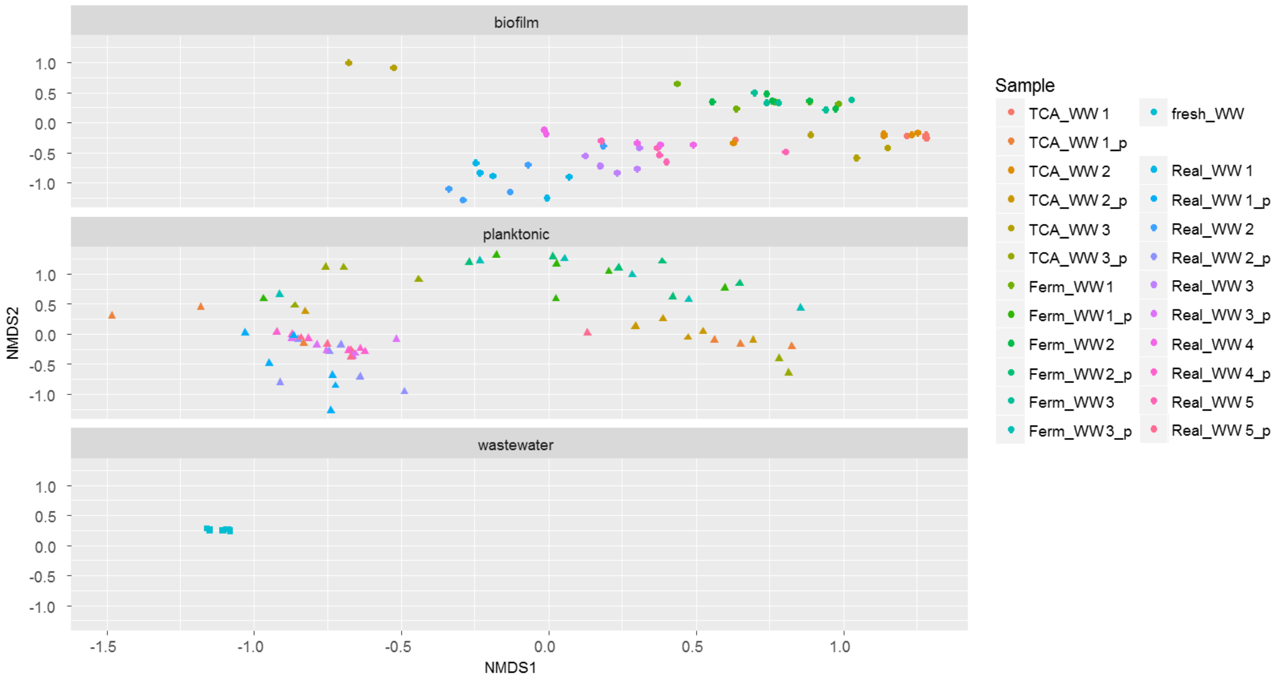
biofilm
planktonic
wastewater
NMDS2
NMDS1
1.0
0.5
0.0
-0.5
-1.0
-1.5
Sample
TCA_WW 1
TCA_WW 1_p
TCA_WW 2
TCA_WW 2_p
TCA_WW 3
TCA_WW 3_p
Ferm_WW 1
Ferm_WW 1_p
Ferm_WW 2
Ferm_WW 2_p
Ferm_WW 3
Ferm_WW 3_p
fresh_WW
Real_WW 1
Real_WW 1_p
Real_WW 2
Real_WW 2_p
Real_WW 3
Real_WW 3_p
Real_WW 4
Real_WW 4_p
Real_WW 5
Real_WW 5_p

C

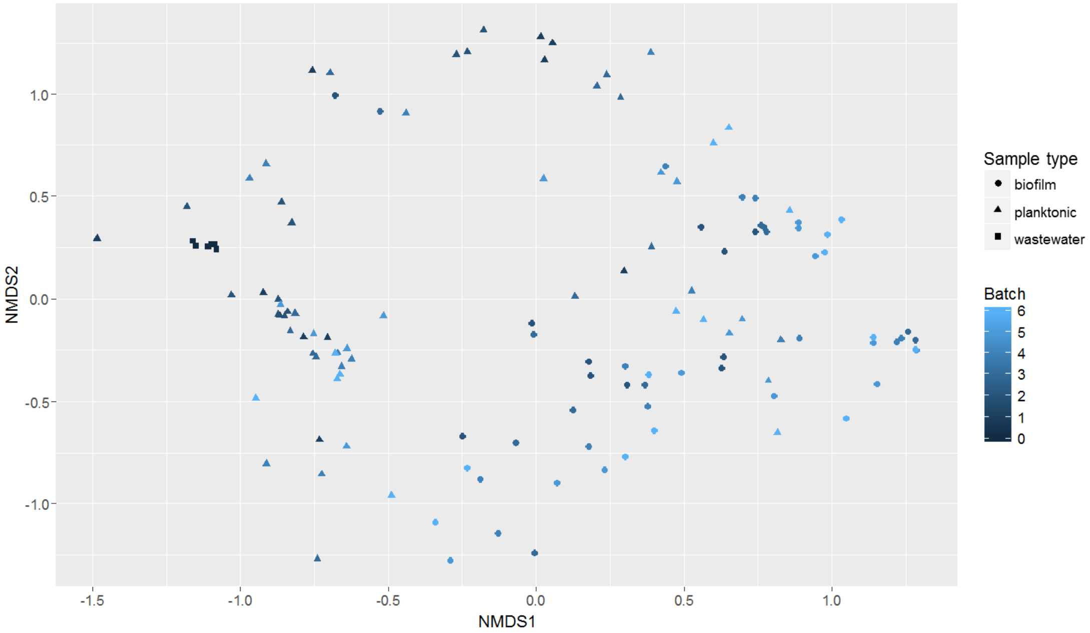

Supplementary Figure 1: Microbial community composition in all samples based on non-metric multidimensional scaling. The color code and the shape of the symbols represent the different wastewaters running with real (Real_WW) or defined wastewater (TCA_WW and Ferm_WW) as well as the sample origin being the fresh domestic wastewater, biofilm or planktonic phase. All samples were used to generate the NMDS plot and then split into the three layers (A) including also a more detailed assignment of the individual reactors (B) with "_p" indicating the samples of the planktonic phase. The color code in (C) represents the time point of sampling regarding the respective batches.

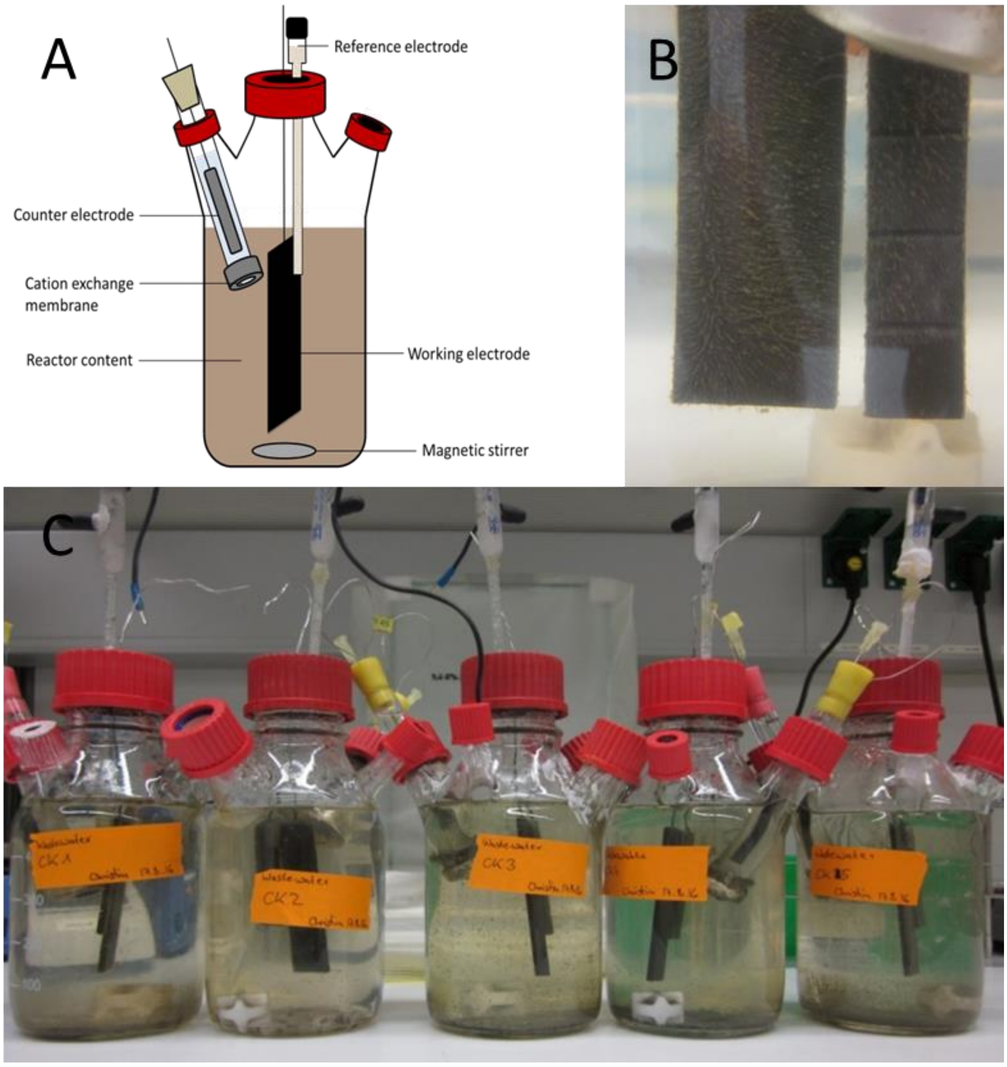

Supplementary Figure 2: Schematic reactor setup (A), representative anode of a Real_WW reactor after batch I, please note the predefined braking points for biofilm sampling (B) and Real_WW reactors 1-5 after batch III (C).

Supplementary Methods: Chemical analysis

Abiotic parameters of the fresh and treated real WW were determined using assay kits NANOCOLOR® CSB 1500 for COD, NANOCOLOR® TOC 600 for TOC, NANOCOLOR® Sulfate 200 for sulfate, NANOCOLOR® TNb 220 for total nitrogen, NANOCOLOR® Nitrate 8 for nitrate, NANOCOLOR® Ammonium 100 for ammonium, NANOCOLOR® *ortho*-and total-Phosphate 45 (NANOCOLOR®, Macherey-Nagel GmbH & Co. KG, Düren, Germany). Conductivity was determined using a Seven Excellence S470 conductivity meter (Mettler-Toledo, Gießen, Germany) and pH was determined using a pH 211, Microprocessor pH Meter (Hanna Instruments, Vöhringen, Germany), following the manufacturer's instructions. The fresh real WW was measured in triplicates and the treated reactor samples once for each reactor and batch. TCA_WW and Ferm_WW were analyzed by HPLC using a HiPlex H-column 300 x 7.7 mm (Agilent Technologies, Inc. CA, USA) with a SecurityGuard Cartridge Carbo-H 4 x 3.0 mm pre-column (Phenomenex, USA) and refractive index detector (RID-10A, Shimadzu Europa GmbH, Duisburg, Germany). The chromatograms were obtained using isocratic elution by the liquid phase of 0.01 N sulfuric acid at a flow rate of 0.5 mL min^{-1} for 50 minutes at 50°C. Prior to HPLC analysis the samples were centrifuged for 10 minutes with 14,000 g at 4°C and subsequently filtered with 0.2 µm nylon filters. Peak identification and calibration (R^2>0.99) were carried out with external standards for sucrose, acetate, propionate and *n*-butyrate.

Supplementary Table 3: Chemical reactions of the anodic half-cell. The following chemical reactions were assumed for the available substrates (assuming neutral charge) in the anodic half-cell and the corresponding number of electrons was used for the respective calculations of Coulombic efficiencies.

Chemical reaction	Number of electrons
$CH_3COOH + 2\ H_2O \rightarrow 2\ CO_2 + 8\ H^+ + 8e^-$	8
$C_3H_6O_2 + 4\ H_2O \rightarrow 3\ CO_2 + 14\ H^+ + 14\ e^-$	14
$C_4H_8O_2 + 6\ H_2O \rightarrow 4\ CO_2 + 20\ H^+ + 20\ e^-$	20
$C_{12}H_{22}O_{11} + 13\ H_2O \rightarrow 12\ CO_2 + 48\ H^+ + 48\ e^-$	48
COD-conversion:<br>Oxidation of 1 g COD	0.125

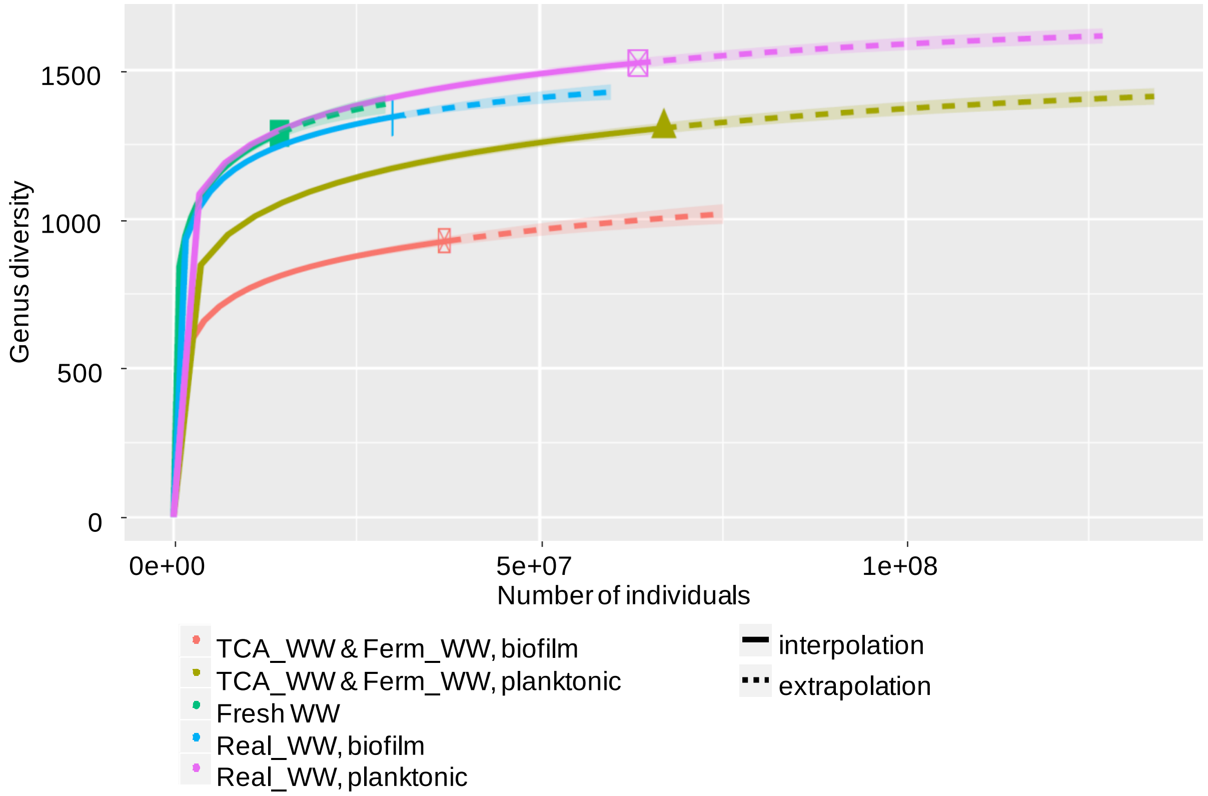

Supplementary Figure 3: Rarefaction curves of OTUs based on sequence reads, separated according the different reactors running with real (Real_WW) or defined wastewater (TCA_WW and Ferm_WW). Samples of the biofilm and the planktonic phase are distinguished.
